# Age-related changes in circulating immune factors reveal biomarkers of immunosenescence

**DOI:** 10.3389/fmed.2026.1729112

**Published:** 2026-01-22

**Authors:** Xin Zhang, Shenjie Xu, Lu Ye, Jingping Yin, Minjie Zhang, Yifeng Jin, Jie Li

**Affiliations:** 1 Department of General Practice, The First Affiliated Hospital of Soochow University, Suzhou, Jiangsu, China; 2 Department of Laboratory Medicine, The First Affiliated Hospital of Soochow University, Suzhou, Jiangsu, China

**Keywords:** IL-6, immunosenescence, sCD28, soluble factors, sPD-1

## Abstract

**Background:**

Immunosenescence, characterized by the decline and restructuring of immune system components with age, affects both innate and adaptive immunity. The predictive value of soluble factors in immunosenescence remains unclear.

**Objective:**

To investigate the predictive value of sCD28 (Soluble CD28), sCD40L (CD40 Ligand), sCD25 (Soluble CD25), IL-6 (Interleukin-6) and sPD-1 (Soluble Programmed Cell Death Protein 1) for immunosenescence in a general population, to evaluate their diagnostic potential using ROC curve analysis, including both individual and combined detection efficacy.

**Methods:**

We analyzed 131 healthy individuals across four age groups: young (≤44 years, *n* = 34), middle-aged (45–60 years, *n* = 31), young-old (61–70 years, *n* = 38), and older (>70 years, *n* = 28). Serum levels of sCD28, sCD40L, sCD25, IL-6, and sPD-1 were measured using ELISA. Baseline characteristics and correlations were analyzed using SPSS 25.0. ROC analysis was performed to assess diagnostic potential, with a focus on both individual markers and the combined detection of sPD-1, IL-6, and sCD28.

**Results:**

sPD-1, IL-6, and sCD28 levels positively correlated with age (*p* < 0.05). IL-6 had the highest individual AUC (0.77, 95%CI: 0.69–0.85; sensitivity 71%, specificity 78% at 1.22 pg./mL). Combined detection of sPD-1, IL-6, and sCD28 improved diagnostic performance, with an AUC of 0.82 (95%CI: 0.76–0.90), sensitivity of 70%, and specificity of 80% at a cut-off of 0.43.

**Conclusion:**

sPD-1, IL-6, and sCD28 are associated with immunosenescence and have diagnostic potential, with IL-6 showing the highest individual efficacy. Importantly, their combined detection enhances diagnostic accuracy for immunosenescence, highlighting their synergistic predictive value for immune aging.

## 1 Introduction

Immunosenescence refers to the age-related decline in immune function, primarily driven by thymic atrophy and associated pathophysiological changes (1, 2). A hallmark of this process is the presence of low-grade chronic inflammation, commonly referred to as “inflammaging. Thymic atrophy leads to a reduced production and functional impairment of naïve T lymphocytes, a hallmark of T cell immunosenescence. The decline in T cell function compromises immune surveillance and clearance, while persistent antigenic stimulation and stress further impair immune responses. This continuous immune dysregulation exacerbates chronic inflammation, promoting inflammatory aging and accelerating overall aging processes.

The mechanisms of inflammatory aging (2, 3) can be summarized through several theories: (1) stress theory: pro-inflammatory state helps the older adults manage chronic antigenic stimulation caused by stressors. However, excessive stress leads to heightened pro-inflammatory responses, which contribute to inflammatory aging. (2) autophagy theory: autophagy helps remove harmful substances from cells to maintain stability and metabolism. With aging, the decline in autophagic clearance efficiency contributes to mitochondrial dysfunction, protein accumulation, elevated reactive oxygen species (ROS) levels, and NLRP3 (4) inflammasome activation, ultimately triggering an inflammatory cascade that accelerates the aging process. (3) DNA damage theory: errors in DNA replication or translation lead to ongoing DNA damage responses (DDR) in telomeric and mitochondrial DNA, promoting cellular aging. DDR activation in senescent cells enhances the pro-inflammatory secretory phenotype, which exacerbates inflammatory aging. (4) stem cell aging theory: stem cell aging is central to overall aging. Pro-inflammatory factors induce the release of inflammatory cytokines, which impair stem cell regeneration, disrupt their function, and compromise the stem cell microenvironment, thereby accelerating the aging process. (5) cytokine theory (5): this theory highlights the significant role of pro-inflammatory cytokines in chronic inflammation, which drives inflammatory aging. Elevated levels of pro-inflammatory cytokines in the bloodstream result in an imbalance between pro-inflammatory and anti-inflammatory processes. The accumulation of these factors creates an inflammatory environment in tissues and organs, affecting immune surveillance and response to treatments. These mechanisms together contribute to the process of inflammatory aging, impacting organ function and immune system efficiency.

Inflammatory aging plays a role in the initiation, progression, malignant transformation, invasion, and metastasis of tumors, making it a crucial component of the tumor microenvironment. For instance, immune checkpoint blocking (ICB) antibody therapies have been widely applied in clinical settings as interventions targeting inflammatory aging. Research (6) indicates that preventive interventions with anti-inflammatory drugs, such as aspirin, can reduce the incidence and mortality of colorectal cancer, suggesting that modulating the cancer-related inflammatory microenvironment may have potential preventive and therapeutic effects on cancer. Therefore, assessing immunosenescence, timely intervention, and restoring immune youthfulness have become major research topics. Currently, the commonly used biomarker for evaluating immunosenescence is IRP (7), IRP typically includes an inverted CD4/CD8 ratio (>1), accumulation of CD8 + CD28 − T cells, and elevated levels of pro-inflammatory cytokines such as IL-6 and TNF-α; most of these parameters worsen with chronological age. IRP may be involved in regulating pyroptosis, a form of programmed cell death critical for eliminating infected or damaged cells. With aging, this clearance mechanism may become less effective, compromising immune responses (8). Given its association with inflammation and pyroptosis, IRP could serve as a biomarker for immune aging. Monitoring IRP levels may help predict susceptibility to infections and diseases, as well as vaccine responsiveness. However, the specific role and mechanisms of IRP in immune aging remain insufficiently explored. Further research is needed to elucidate its functions and optimize its use for immune aging assessment and intervention. Other soluble markers, such as TNF-*α* and IFN-*γ*, are also commonly used as biomarkers for immunosenescence detection, but their specificity is relatively low, as they can also be detected during acute inflammation (9, 10).

In recent years, immune molecules, particularly membrane-bound and soluble molecules upregulated during T cell activation and effector function, have been identified as key players in immunosenescence and potential biological markers for its assessment. Recent studies have highlighted profound age-associated alterations in T lymphocyte compartments, particularly the accumulation of senescent CD28^−^CD27^−^ T cell subsets. This demonstrated that both CD8^+^CD28^−^CD27^−^ and CD4^+^CD28^−^CD27^−^ T cells significantly increase with aging, reflecting progressive immune senescence (11–13). Studies have suggested that sCD163, sCTLA-4, sCD80, and sCD28 are potential soluble markers for detecting immunosenescence (14).

The relevant factors involved in our study are as follows: sCD28 (15) facilitates T-cell costimulation, with elevated levels linked to chronic inflammation and T-cell dysfunction. CD40L (16) promotes B-cell activation and antibody production, modulating immune responses. sCD25 servesas a marker of T-cell activation, associated with inflammation and autoimmune disorders. IL-6 (17), a pro-inflammatory cytokine, plays a key role in chronic inflammation and immune dysregulation. It has been demonstrated (18) that IL-6 promotes the differentiation of Th17 cells in combination with transforming growth factor (TGF)-*β*, while simultaneously inhibiting TGF-β-induced differentiation of regulatory T cells (Treg). The upregulation of the Th17/Treg balance is associated with the development of chronic inflammation. sPD-1 (16) modulates the PD-1/PD-L1 pathway, influencing T-cell responses, tumor immunity, and autoimmunity. These factors collectively regulate immune activation and function. With aging, their dysregulated expression contributes to chronic inflammation, immune decline, and increased disease susceptibility, marking immune aging.

This study primarily investigates the levels of inflammatory cytokines in different age groups to better understand the process of immune aging. Soluble factors such as sCD28, CD40L, sCD25, IL-6, and sPD-1 are selected as research subjects. Changes in the expression of soluble factors across normal populations of different ages are analyzed to identify biomarkers predictive of immune aging, offering potential early warning and preventive measures for immune aging-related diseases.

## 2 Materials and methods

### 2.1 Study subjects and grouping

A total of 131 healthy individuals (78 males, 59.5%; 53 females, 40.5%) undergoing routine health examinations at the First Affiliated Hospital of Soochow University between May 2024 and February 2025 were included. Anthropometric data, including body mass index (BMI), waist circumference, and heart rate, were collected using standardized procedures.

Participants were stratified into four age groups based on the United Nations “older persons” classification framework, with minor adjustments aligned with prior immunosenescence research: young (≤44 years, *n* = 34; mean age: 35.9 ± 5.8 years), middle-aged (45–60 years, *n* = 31), young-old (61–70 years, *n* = 38), and older (>70 years, *n* = 28). While emerging evidence suggests that immune system alterations may initiate as early as 25 years of age, the present stratification was selected for consistency with widely accepted gerontological and immunological cohort comparisons, which facilitates cross-study validation of age-related immune parameter trends. Serum concentrations of soluble immune-aging biomarkers, including sCD28, sCD40L, sCD25, IL-6, and sPD-1, were quantified and compared across all age cohorts to characterize age-dependent immunological shifts.

This retrospective observational study was approved by the Ethics Committee of the First Affiliated Hospital of Soochow University (Approval No. 2025166, February 28, 2025). Written informed consent was obtained from all participants.

### 2.2 Inclusion and exclusion criteria

Participants of all age groups who underwent routine health examinations and were deemed generally healthy based on clinical evaluation and laboratory testing were eligible for inclusion in this study.

1. Participants with a history of malignancy, autoimmune or immunodeficiency disorders, or any other condition known to affect immune function were excluded. Individuals with clinically significant cardiovascular diseases—such as symptomatic coronary artery disease, congestive heart failure of New York Heart Association (NYHA) class II or above, severe arrhythmias requiring treatment, or myocardial infarction within the previous 12 months—were also excluded.
2. Pregnant or lactating women and those who had received hormone therapy within the preceding 3 months were not eligible due to potential hormonal effects on circulating soluble factors. Subjects who had recently received immunosuppressive therapy or presented with active infections were likewise excluded. Infection-free status was verified by negative hepatitis B surface antigen/antibody, anti-HCV, anti-HIV, anti-TP, and C-reactive protein (CRP) tests.
3. Participants with major hematologic, renal, metabolic, gastrointestinal, or endocrine dysfunctions, or other severe uncontrolled comorbidities, were also excluded. In addition, individuals with congenital or acquired immune deficiencies, a history of organ transplantation, or long-term corticosteroid use for immune-related conditions were not included in the analysis.

### 2.3 Research methods

Fasting venous blood samples (5 mL; ≥8 h fast) were collected from all participants between 08:00 and 10:00 a.m. on the morning following admission to minimize circadian variation. The blood was allowed to clot at room temperature for 1 h and subsequently centrifuged at 3000 rpm for 10 min. The resulting serum was aliquoted into 500 μL portions and stored at −80 °C until analysis.

Serum concentrations of sCD28, sCD40L, sCD25, IL-6, and sPD-1 were quantified using enzyme-linked immunosorbent assays (ELISA) according to the manufacturer’s protocols (Suzhou Xuguang Kexing Antibody Biotechnology Co., Ltd., Suzhou, China). The assays demonstrated linearity over the following ranges: sCD28 (0.2–30 ng/mL), sCD40L (0.5–50 ng/mL), sCD25 (0.1–20 ng/mL), IL-6 (1.5–300 pg./mL), and sPD-1 (0.3–40 ng/mL). The intra-assay and inter-assay coefficients of variation were <6 and <10%, respectively.

### 2.4 Statistical methods

Statistical analysis was performed using SPSS 25.0 software. The normality of the data was assessed using the Shapiro--Wilk test. Continuous variables were expressed as mean ± standard deviation (SD), while categorical variables were presented as frequencies and percentages. Independent sample *t*-tests were used to compare continuous variables between two groups, and chi-square tests were used for categorical variables. Spearman’s correlation coefficient was used for correlation analysis. Multiple linear regression analysis was employed to evaluate the effect of multiple independent variables on a continuous dependent variable. Additionally, ROC curve analysis was conducted to assess the diagnostic potential of sPD-1, IL-6, sCD28, sCD40L, and sCD25 for immunosenescence. The area under the ROC curve (AUC) was calculated, and the optimal cut-off values, sensitivity, and specificity were determined. All tests were two-tailed, and *p* < 0.05 was considered statistically significant.

### 2.5 Ethical considerations

All methods were performed in accordance with the relevant guidelines and regulations. The study was approved by the Ethics Committee of The First Affiliated Hospital of Soochow University (Approval Number: 2025166, Date: February 28, 2025). According to the approved protocol, participant recruitment and data collection were conducted between May 2024 and February 2025, as specified in the ethical approval document. The study was conducted in accordance with the principles of the Declaration of Helsinki. Informed consent was obtained from all participants or their legal representatives.

## 3 Result

### 3.1 The baseline data analysis showed no statistical difference

As showed in Tables 1, 131 participants were included in the study and categorized into four age groups: Y group, M group, YO group, O group. No significant differences were observed in gender, BMI, heart rate, or waist circumference across the four groups (*p* > 0.05).

**Table 1:** Comparison of basic characteristics among the four study groups.

Indicator	Y group (*n* = 34)	M group (*n* = 31)	YO group (*n* = 38)	O group (*n* = 28)	F(X^2^)	*p* value
Male [case (%)]	20 (60.6%)	15 (46.9%)	26 (70.3%)	17 (58.6%)	3.925	0.27
BMI (Kg/m^2^)	25.35 ± 3.94	23.76 ± 2.87	24.21 ± 2.21	24.63 ± 3.27	372.57	0.438
HR(times/min)	76.52 ± 9.75	79.62 ± 11.33	74. 19 ± 8.45	77.93 ± 11.15	119.211	0.35
WC (cm)	80.30 ± 12.45	79.44 ± 9.07	84.76 ± 8.16	84.45 ± 10.78	135.393	0.159

F(X^2^) denotes F-statistic or Chi-square value; BMI: body mass index; HR: heart rate; WC: waist circumference. Data collected on the day of blood sampling.

### 3.2 Age increase correlates with soluble molecule changes

**Figure 1:**
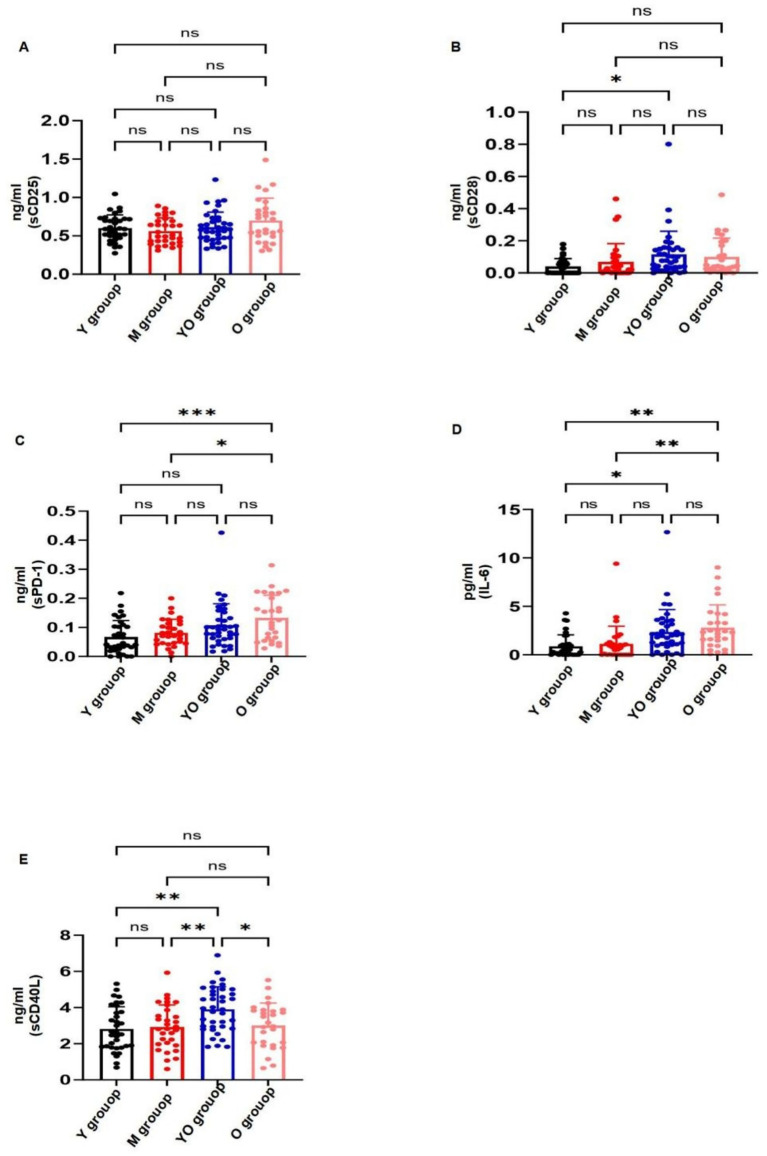
Age-related trends in soluble factor levels across the four age groups. **(A)** Changes in sCD25 levels across different age groups. The figure illustrates the levels of sCD25 in the Young (Y), Middle-aged (M), Young-old (YO), and Older (O) groups. Data are presented in ng/mL. *p* = 0.088indicating that the differences in sCD25 levels among the different age groups did not reach statistical significance. **(B)** Changes in sCD28 levels across different age groups. The figure illustrates the levels of sCD28 in the Young (Y), Middle-aged (M), Young-old (YO), and Older (O) groups. Data are presented in ng/mL. *p* = 0.016 indicates a significant difference compared to the control group. **(C)** Changes in sPD-1 levels across different age groups. The figure illustrates the levels of sPD-1 in the Young (Y), Middle-aged (M), Young-old (YO), and Older (O) groups. Data are presented in ng/mL. *p* = 0.027 indicates a significant difference compared to the control group. **(D)** Changes in IL-6 levels across different age groups. The figure illustrates the levels of IL-6 in the Young (Y), Middle-aged (M), Young-old (YO), and Older (O) groups. Data are presented in pg./mL. *p* = 0.006 indicates a significant difference compared to the control group. **(E)** Changes in sCD40L levels across different age groups. The figure illustrates the levels of sCD40L in the Young (Y), Middle-aged (M), Young-old (YO), and Older (O) groups. Data are presented in ng/mL. *p* = 0.048 indicates a significant difference compared to the control group. *Indicates a statistically significant difference (usually *p* < 0.05) between groups. **Indicates a highly statistically significant difference (usually *p* < 0.01) between groups. ns: Indicates “no significant difference” (i.e., *p* > 0.05) between groups.

With advancing age, serum sCD25 levels showed an upward trend, reaching the highest values in the older (O) group, although the differences among age groups were not statistically significant. In contrast, sCD28, sPD-1, and IL-6 exhibited clear age-related increases. Specifically, sCD28 differed significantly between the young (Y) and young-old (YO) groups, sPD-1 between the Y and O groups, and IL-6 between both the Y and O groups and the middle-aged (M) and O groups. These results suggest that sCD28, sPD-1, and IL-6 may serve as potential biomarkers of immunosenescence. Interestingly, sCD40L levels increased up to approximately age 60 and then declined thereafter, with significant differences observed between the Y and YO groups and between the M and YO groups, the biphasic pattern of sCD40L with age may reflect an early compensatory activation of the CD40/CD40L pathway, followed by immune exhaustion or downregulation of T-cell helper activity in advanced age (see Figure 1).

### 3.3 Age linked to sCD28, sPD-1, IL-6, sCD40L levels

Correlation analysis demonstrated significant positive associations between age and the soluble immune factors sPD-1, IL-6, and sCD28, indicating progressive immune activation and inflammatory remodeling with advancing age. These findings suggest that upregulation of immune checkpoint (sPD-1), pro-inflammatory (IL-6), and co-stimulatory (sCD28) pathways collectively reflects the molecular signatures of immunosenescence. In contrast, sCD25 and sCD40L showed no significant correlations with age, implying that IL-2 receptor shedding and CD40/CD40L signaling may not be major contributors to physiological immune aging under healthy conditions (Table 2).

**Table 2:** Correlation between age and sCD25, sCD28, sPD-1, IL-6, sCD40L.

Indicator	Age	
	*r*	*p* value
sPD-1	0.35	<0.00 L*
IL-6	0.451	<0.001*
sCD28	0.313	<0.001*
sCD25	0.086	0.329
sCD40L	0.157	0.073

“r” means correlation coefficient value, *p* value: probability value *p < 0.01.

Multivariate linear regression identified age as an independent predictor of several soluble immune factors. Significant associations were observed between age and sCD28, sPD-1, IL-6, and sCD25, with standardized regression coefficients (*β*) of 0.002, 0.002, 0.050, and 0.002, respectively (all *p* < 0.05). These results indicate that advancing age is accompanied by enhanced expression of molecules involved in immune co-stimulation (sCD28), immune checkpoint regulation (sPD-1), and systemic inflammation (IL-6), reflecting the coordinated remodeling of immune signaling pathways during immunosenescence. Detailed results are summarized in Table 3.

**Table 3:** Multivariate linear regression analysis of age and sCD25, sCD28, sPD-1, IL-6, sCD40L.

Dependent variable	*β*	Beta	*t*	*p* value
SPD-1	0.002	0.342	4.129	0.000*
IL-6	0.05	0.365	4.455	0.000*
sCD28	0.002	0.246	2.885	0.005*
sCD25	0.002	0.135	1.549	0. 124
sCD40L	0.015	0.174	2.002	0.047*

β denotes Non-standardized regression coefficients, Beta denotes standardized regression coefficients, t denotes *t*-statistics, *p* value: probability value, *p < 0.05.

### 3.4 ROC analysis results for immune senescence biomarkers

Participants aged ≤60 years were classified as non-senescent and those >60 years as senescent, consistent with the threshold at which age-related physiological and immune alterations commonly emerge. Logistic regression models were applied to estimate the probability of immunosenescence, and receiver operating characteristic (ROC) curve analysis was performed to evaluate the diagnostic performance of each soluble factor.

**Figure 2:**
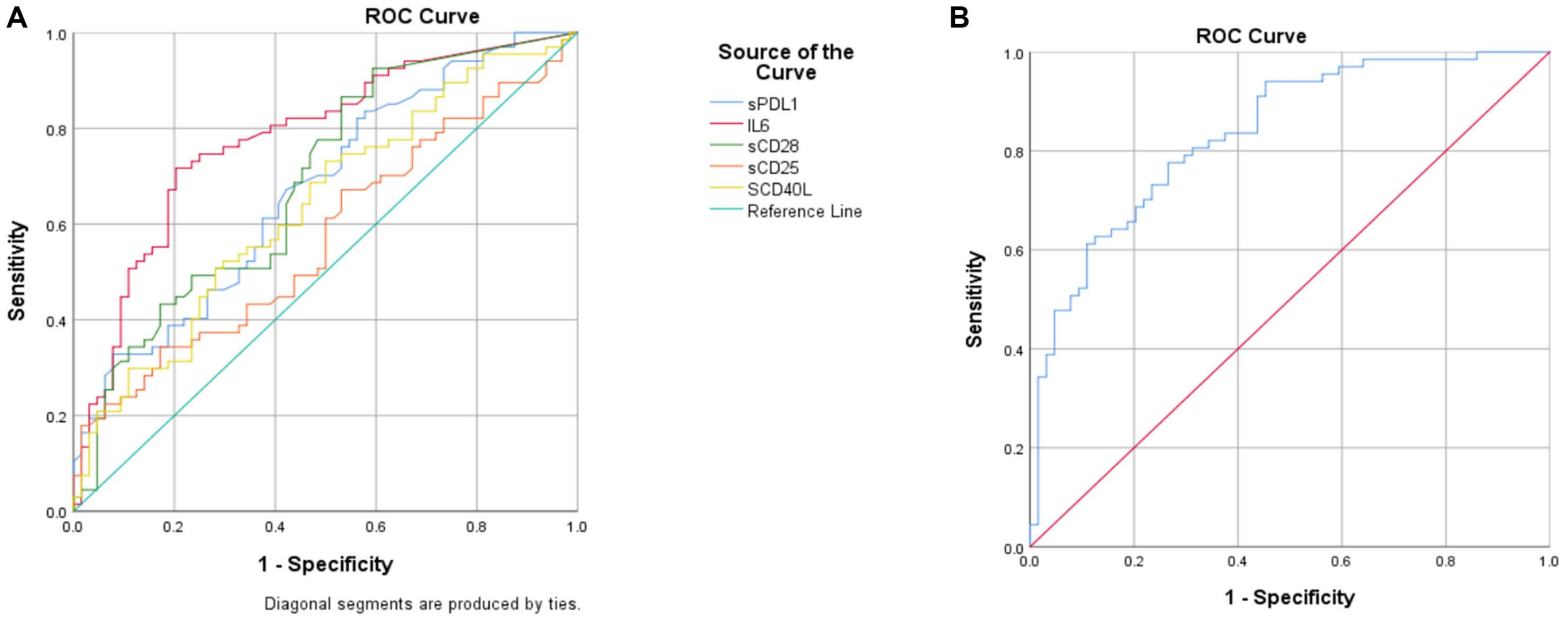
ROC curves and areas under the curve (AUCs) for individual and combined soluble factors. Graph A shows multiple curves for sPDL1, IL6, sCD28, sCD25, and sCD40L, with a reference line for comparison. Graph B shows ROC curve for the combined prediction of IL-6, sPD-1, and sCD28.

IL-6, sPD-1, and sCD28 demonstrated the strongest discriminative ability, with higher AUC values indicating superior predictive performance (Figure 2; Table 4). The combined detection of these markers yielded the highest AUC, reflecting the complementary roles of chronic inflammation (IL-6), T-cell co-stimulation (sCD28), and immune checkpoint dysregulation (sPD-1) in the development of immunosenescence.

**Table 4:** ROC analysis results for immune senescence biomarkers.

Indicator	AUC (95%CI)	Cut-off value	Sensitivity	Specificity
sPD-1	0.67 (0.58–0.76)	0.15 ng/mL	33%	92%
IL-6	0.77 (0.69–0.85)	1.22 pg./mL	71%	78%
sCD28	0.68 (0.59–0.77)	0.01 ng/mL	86%	46%
sCD40L	0.65 (0.55–0.74)	2.77 ng/mL	74%	51%
sCD25	0.56 (0.46–0.67)	0.90 ng/mL	16%	98%
Combined detection	0.82(0.76–0.90)	0.43	70%	80%

AUC, area under the receiver operating characteristic curve; CI, confidence interval. Cut-off values were determined using the Youden index to maximize sensitivity and specificity. Combined detection refers to the joint analysis of sPD-1, IL-6, and sCD28.

## 4 Discussion

It is important to distinguish chronological aging from biological immunosenescence. While chronological age reflects the passage of time, immunosenescence represents a cumulative biological process characterized by defined cellular and molecular hallmarks, including T-cell exhaustion, reduced naïve T-cell output, and the senescence-associated secretory phenotype (SASP). Therefore, the present analysis interprets soluble factor alterations not merely as age-associated variations but as potential molecular indicators of biological immune aging. This study is the first to systematically explore the relationship between sCD28, sCD40L, sCD25, IL-6, sPD-1, and age in healthy individuals. We found that sPD-1, sCD28, IL-6levels rise with age in healthy elderly people, providing new insights into its role in immune senescence in non-pathological contexts.

Significant positive correlations were observed between serum levels of sPD-1, IL-6, and sCD28 and chronological age (*r* = 0.35, 0.451, and 0.313, respectively), indicating progressive immune activation and dysregulation with aging. These associations align with established mechanisms of immunosenescence, in which chronic antigenic stimulation and sustained low-grade inflammation—collectively known as inflammaging—promote the upregulation of soluble immune modulators.

IL-6, a central cytokine in the inflammatory network, is widely recognized as a key mediator linking systemic inflammation to age-related immune decline. Elevated IL-6 levels reflect enhanced activation of the NF-κB and STAT3 pathways, which sustain a pro-inflammatory milieu and drive the senescence-associated secretory phenotype (SASP). Similarly, increased circulating sPD-1 may represent an adaptive response to chronic immune activation, reflecting T-cell exhaustion and checkpoint pathway dysregulation. The rise in soluble CD28 (sCD28) likely mirrors compensatory T-cell co-stimulatory signaling in response to declining membrane-bound CD28 expression, a hallmark of aging T-cell populations.

In contrast, sCD25 levels showed no significant association with age (*r* = 0.086), suggesting that IL-2 receptor shedding is not a dominant feature of physiological immune aging and may instead occur under pathological or acute inflammatory conditions. Although sCD40L exhibited only a weak, non-significant positive correlation with age (*r* = 0.157), the observed upward trend could indicate early subclinical activation of the CD40/CD40L axis, warranting further verification in larger and more diverse cohorts.

Together, these findings highlight that the gradual elevation of sPD-1, IL-6, and sCD28 reflects the cumulative impact of persistent immune activation, loss of T-cell homeostasis, and the transition toward an inflammaging phenotype. These soluble factors thus provide a minimally invasive molecular signature for tracking the progression of immune aging in healthy populations.

Notably, sCD25 levels remained stable across age groups, suggesting that its regulation is more influenced by acute immune activation or homeostatic feedback rather than chronological aging. Additionally, sCD40L exhibited an inverted U-shaped trajectory, increasing from young to middle-aged groups and then declining in the elderly, which may reflect an initial compensatory upregulation of helper T-cell activity followed by exhaustion or downregulation in advanced age. Longitudinal studies with larger samples are needed to determine whether these patterns are true age-dependent changes or cohort-specific effects.

ROC analysis underscores the integrated diagnostic value of IL-6, sPD-1, and sCD28 in identifying immunosenescence. The combined model achieved an AUC of 0.82, markedly outperforming individual markers and highlighting the multifactorial nature of immune aging. This improvement likely stems from the complementary biological roles of these molecules—IL-6 representing systemic inflammaging, sPD-1 reflecting T-cell exhaustion and checkpoint activation, and sCD28 indicating the loss of co-stimulatory balance in aging lymphocytes. Their synergistic detection captures both inflammatory and regulatory dimensions of immunosenescence, offering a more comprehensive and accurate assessment than single biomarkers. Nevertheless, these results should be interpreted with caution. Circulating cytokine and receptor levels are influenced by multiple factors, including metabolic status, subclinical inflammation, and undiagnosed comorbidities. Thus, their observed changes may reflect systemic physiological variation rather than direct causal mechanisms of immunosenescence. Clinically, these soluble markers may be more suitable for immune risk stratification or longitudinal monitoring rather than definitive diagnosis.

Previous studies have also shown that reduced expression of sCD28 in the older adults is associated with T cell dysfunction (19), which is consistent with the findings of this study. However, some studies have reported a decrease in CD40L (20) levels in certain chronic inflammatory diseases, which is associated with the heterogeneous effects of disease states on immune cells. The mechanism of action of sCD25 in immunosenescence is still not fully understood. This study provides evidence of its age-related changes in a normal population, laying the foundation for further research on its role in immune regulation. IL-6, as a key factor in chronic inflammation, has been widely recognized for its role in immunosenescence. This study further confirms its predictive value in healthy older adults populations. Research on sPD-1 is relatively limited, but existing studies suggest its role in tumor immune evasion. This study broadens its research perspective in the field of immunosenescence.

The results of this study offer potential biomarkers for evaluating immune aging in the older adults, which could facilitate the early detection of individuals with declining immune function, thus enabling targeted immunotherapeutic interventions. Future research should aim to broaden sample sizes to encompass a diverse range of regions and populations, while also exploring the distinct roles these soluble factors play in various chronic diseases. Moreover, the integration of genomics, proteomics, and other omics technologies could provide deeper insights into the specific molecular mechanisms by which these factors influence immune aging, forming the basis for the development of innovative immunomodulatory drugs and therapeutic approaches.

Additionally, exploring potential interventions targeting these soluble factors, such as modulating their expression levels or inhibiting their signaling pathways, holds promise for delaying the progression of immune aging and improving the quality of life and health outcomes in older adults populations. This area of research represents a vital direction for future studies. The potential pathways that may exist are listed below and can be used as a reference to explore their specific roles in immunosenescence:

sCD28 (21, 22) is a co-stimulatory molecule expressed on T cells, and its soluble form may influence T cell activation and proliferation. *In vitro*, sCD28 can stimulate T cell proliferation and induce the secretion of pro-inflammatory cytokines such as IL-6 and TNF-α. The elevation of these pro-inflammatory cytokines is a characteristic feature of chronic low-grade inflammation. By promoting the secretion of these factors, sCD28 exacerbates the state of chronic low-grade inflammation. As a soluble form of the T cell co-stimulatory molecule, sCD28 can competitively bind to CD28 or CTLA-4, disrupting their interaction with B7 molecules. This disruption may lead to abnormal activation of immune cells, thereby triggering or aggravating chronic low-grade inflammation. Changes in sCD28 levels with aging may be associated with a decline in T cell function, which in turn influences the progression of immune aging.

CD40L plays a crucial role in various autoimmune diseases, such as systemic lupus erythematosus (SLE) and rheumatoid arthritis (RA). These diseases are often characterized by chronic low-grade inflammation. CD40L (23) enhances the activation of B cells and T cells by binding to CD40, promoting the production of inflammatory mediators such as TNF and IL-1β, thus driving the progression of chronic low-grade inflammation. During the inflammatory process, the interaction between CD40L and CD40 activates a variety of inflammatory cells, including macrophages and dendritic cells. The activation of these cells results in the release of a large amount of pro-inflammatory factors, which sustain and exacerbate the chronic low-grade inflammatory state, influencing antibody production and antigen presentation. These processes are closely associated with immune aging.

sCD25 is the *α* chain of the IL-2 receptor (24, 25), and its soluble form may influence T cell responses to IL-2. IL-2 plays a critical role in immune regulation, and an increase in sCD25 levels could lead to immune dysregulation, impairing the immune system’s ability to control inflammation and thus promoting the onset and progression of chronic low-grade inflammation. Elevated CD25 levels may affect T cell function, resulting in aberrant T cell responses to inflammation, which further exacerbates the chronic low-grade inflammatory state.

IL-6 is a multifunctional cytokine involved in both inflammatory responses and immune regulation (18). IL-6 is a key pro-inflammatory cytokine that plays a central role in chronic low-grade inflammation. It promotes the proliferation and differentiation of inflammatory cells, increases the production of other pro-inflammatory factors, and triggers a cascade of inflammatory mediators, thereby exacerbating chronic low-grade inflammation. Sarcopenia is a major age-related phenotype closely linked to chronic low-grade inflammation and immune dysregulation. Pro-inflammatory cytokines such as IL-6 contribute to muscle catabolism, suggesting that immunosenescence and musculoskeletal decline may share common inflammatory mechanisms (26, 27). IL-6 regulates the expression of various inflammatory mediators, such as CRP and TNF-α, through activation of downstream signaling pathways like signal transducer and activator of transcription 3 (STAT3). These mediators collectively maintain the state of chronic low-grade inflammation. Elevated IL-6 levels with aging are associated with chronic low-grade inflammation and may accelerate the progression of immune aging. Beyond its pro-inflammatory role, IL-6 can exert anti-inflammatory effects via trans-signalling through the soluble IL-6 receptor, a mechanism that may become dysregulated with ageing (28).

sPD-1 is the soluble form of PD-1, and the PD-1/PD-L1 signaling pathway (29, 30) plays a crucial role in immune suppression. sPD-1 may regulate immune cell activity by modulating this signaling pathway, leading to an imbalance between immune suppression and inflammatory responses, thereby affecting the development of chronic low-grade inflammation. Increased levels of sPD-1 may be associated with T cell exhaustion, which results in a decline in immune surveillance, making it difficult to control inflammatory responses effectively and further exacerbating chronic low-grade inflammation. Recent studies show an age-related accumulation of senescent CD28^−^CD27^−^ T-cell subsets and increased T-cell exhaustion. In this context, elevated sCD28 and sPD-1 may serve as indirect, minimally invasive surrogates of cellular immunosenescence, consistent with current literature (14).

Classical assessment of immunosenescence relies on well-established cellular markers, Future studies integrating cellular immunophenotyping (e.g., CD4/CD8 inversion, CD28^−^CD27^−^ accumulation) will help clarify whether soluble markers such as IL-6, sPD-1, and sCD28 act as drivers or downstream correlates of immunosenescence including inversion of the CD4/CD8 ratio, loss of CD28 and CD27 expression on T cells, and the accumulation of highly differentiated or senescent T-cell subsets. These parameters constitute the core of the Immune Risk Profile and reflect fundamental alterations in T-cell homeostasis with aging. Although these cellular markers were not measured in the present study-such as CD28/CD27 loss on T cells or CD4/CD8 ratio inversion—were not directly assessed in this study. Therefore, the soluble immune factors analyzed here should be interpreted as complementary or surrogate indicators of immune aging rather than direct cellular hallmarks, they provide an essential reference framework for interpreting age-related changes in soluble immune factors (11).

This study has several limitations. Firstly, the sample size is relatively small, and the majority of participants are from the Suzhou region, which may introduce regional bias into the results. Secondly, this study did not measure classical immune senescence markers (such as the CD4/CD8 ratio and SASP factors), and thus could not directly compare the efficacy of soluble factors with cellular immune markers. Future research should validate these findings by integrating multidimensional indicators. Furthermore, the study did not explore the interactions between these factors and their synergistic effects with other immune cells and molecules, which may represent an important direction for future research. Additional limitations include lack of sex-stratified analysis, body-composition data, dietary habits.

In summary, soluble factors such as sCD28, CD40L, sCD25, IL-6, and sPD-1 are closely linked to chronic low-grade inflammation through various mechanisms. These factors not only serve as biomarkers for chronic low-grade inflammation but also actively contribute to its initiation and progression by modulating immune cell activation and the secretion of inflammatory mediators. Further investigation into the relationship between these soluble factors and chronic low-grade inflammation is crucial for advancing our understanding of the underlying pathological mechanisms and for the development of novel therapeutic strategies. In the future, I will expand the sample size to include multicenter healthy populations, and integrate longitudinal follow-up data to validate the dynamic changes in factor levels. Additionally, I will explore through *in vitro* experiments how sPD-1 regulates T-cell senescence via the PD-1/PD-L1 pathway.

## Data Availability

The original contributions presented in the study are included in the article/supplementary material, further inquiries can be directed to the corresponding authors.
